# Geriatric nutritional risk index predicts perioperative cardiovascular events in older patients with coronary artery disease undergoing non-cardiac surgery: a multicenter retrospective cohort study

**DOI:** 10.3389/fnut.2025.1652742

**Published:** 2025-10-10

**Authors:** Xiaolin Li, Congying Wang, Haodong Jiang, Jia Zhu, Runzhe Wu, Yongquan Niu, Feiyu Chen, Yunpeng Jin

**Affiliations:** ^1^Department of Nutrition, The Fourth Affiliated Hospital of School of Medicine, and International School of Medicine, International Institutes of Medicine, Zhejiang University, Yiwu, Zhejiang, China; ^2^Department of Cardiology, The Fourth Affiliated Hospital of School of Medicine, and International School of Medicine, International Institutes of Medicine, Zhejiang University, Yiwu, Zhejiang, China

**Keywords:** geriatric nutritional risk index, perioperative cardiovascular events, coronary artery disease, non-cardiac surgery, revised cardiac risk index

## Abstract

**Background:**

The relationship between geriatric nutritional risk index (GNRI) and perioperative cardiovascular events (PCE) remains underexplored. This study aimed to evaluate the predictive utility of GNRI for PCEs in older patients with coronary artery disease (CAD) undergoing non-cardiac surgery.

**Methods:**

This multicenter retrospective study analyzed consecutive patients aged ≥ 65 years with documented CAD undergoing non-cardiac surgery between 2013 and 2024 at two Chinese tertiary medical centers. The primary outcome was a composite of PCEs, including death, resuscitated cardiac arrest, myocardial infarction, heart failure, and stroke, occurring intraoperatively or during postoperative hospitalization.

**Results:**

Among 7,272 participants, 408 (5.6%) experienced PCEs. GNRI exhibited a significant inverse linear correlation with PCEs (OR = 0.92; 95% CI: 0.91–0.93; *p* < 0.001). Using a GNRI cutoff of 98, the at-risk group (GNRI < 98) had a significantly higher incidence of PCEs compared to the no-risk group (GNRI ≥ 98) (univariate OR = 4.840; 95% CI: 3.947–5.935; *p* < 0.001; multivariate OR = 1.919; 95% CI: 1.496–2.461; p < 0.001). GNRI demonstrated comparable discriminatory ability to revised cardiac risk index (RCRI) (C-statistics: 0.676 vs. 0.694, *p* = 0.309). A weighted scoring system incorporating GNRI and RCRI significantly outperformed either index alone in predicting PCEs (vs. RCRI: C-statistics 0.768 vs. 0.694, *p* < 0.001; vs. GNRI: C-statistics 0.768 vs. 0.676, *p* < 0.001).

**Conclusion:**

The GNRI independently predicted PCEs in older CAD patients undergoing non-cardiac surgery. Integrating GNRI into clinical decision-making may enhance perioperative risk stratification and management in this high-risk population, though further validation is warranted.

## Introduction

Perioperative cardiovascular events (PCE) are a major cause of morbidity and mortality for over 50 million patients with established coronary artery disease (CAD) undergoing non-cardiac surgery worldwide each year (1). PCEs, including death, cardiac arrest, myocardial infarction, heart failure, and stroke, affect more than 5% of CAD patients undergoing non-cardiac surgery (2). Accurate preoperative cardiovascular risk assessment is essential for optimizing the evaluation and management of this high-risk population (3). Revised cardiac risk index (RCRI) is the most widely used predictive model due to its simplicity and global validation (4). However, its predictive accuracy for PCEs in older Chinese patients with CAD has been shown to be no better than chance (5), highlighting the need for more reliable risk assessment tools in this population.

Geriatric nutritional risk index (GNRI) is a validated tool specifically designed to assess nutrition-related risks of morbidity and mortality in hospitalized older patients (6). Emerging evidence suggests that GNRI is associated with perioperative outcomes in older patients undergoing specific surgical procedures, such as pancreatoduodenectomy, esophageal surgery, and nephrectomy (7–9). Despite these advancements, the role of GNRI in predicting PCEs in older CAD patients remains unexplored.

To address this gap, we conducted a multicenter retrospective analysis to evaluate the predictive value of GNRI for PCEs in older CAD patients undergoing non-cardiac surgery. This study aimed to provide evidence on the potential role of GNRI in improving preoperative risk stratification and management strategies for this high-risk cohort.

## Materials and methods

### Study design and participants

This multicenter retrospective study included consecutive patients aged ≥ 65 years with documented CAD who underwent non-cardiac surgery at two tertiary academic medical centers in Zhejiang, China. Participants were recruited from the First Affiliated Hospital of Zhejiang University School of Medicine (AHZU) between January 1, 2013 and May 31, 2021, and from the Fourth AHZU between October 1, 2020, and October 31, 2024.

This study was adhered to the Declaration of Helsinki and received ethical approval from the Institutional Review Boards (IRB) of both participating institutions. The First AHZU granted approval (Approval No. IIT20230114A; February 2023) for data collection spanning from 2013 to 2021. Subsequently, the Fourth AHZU provided approval (Approval No. K2024222; December 2024) for data collection covering the period from 2020 to 2024. The overlapping data collection period (1 October 2020 to 31 May 2021) falls within the valid approval periods of both institutions. Due to the retrospective nature of the study, the requirement for written informed consent was waived. All data were anonymized and de-identified prior to analysis.

The CAD was defined based on any of the following criteria: angiographic evidence of coronary stenosis > 50%; documented myocardial infarction > 3 months prior to enrollment; coronary revascularization > 3 months prior to enrolment; positive results on myocardial perfusion scintigraphy or exercise stress test; or typical anginal symptoms accompanied by electrocardiographic evidence of myocardial ischemia (10). Surgical procedures included elective non-cardiac surgery, classified according to the American College of Cardiology (ACC)/American Heart Association (AHA) guidelines for perioperative cardiovascular assessment (11). Exclusion criteria comprised: day surgery; emergency surgery; patients who underwent multiple (≥2) operations during a single hospital admission; and incomplete or insufficient clinical data for comprehensive analysis. All patients underwent routine preoperative evaluations following established perioperative management guidelines.

### Data collection

Data were obtained from the integrated electronic medical record systems of the First and Fourth AHZU. Initial patient identification was conducted using the International Classification of Diseases, Tenth Revision (ICD-10) coding system to identify all surgical department discharges with CAD diagnoses during the study period. Each case was manually reviewed and rigorously assessed against the predefined inclusion and exclusion criteria. Clinical data extracted from electronic medical records included demographic characteristics, preoperative evaluations, American Society of Anesthesiologists (ASA) physical status classifications, surgical types, anesthesia techniques, perioperative cardiovascular complications, and other relevant perioperative information. All preoperative assessments were conducted within 30 days preceding surgery. Extraneous data were excluded from analysis. When multiple measurements were available during this period, the temporally closest value to the surgical date was selected to maximize clinical relevance.

### Predictors

Geriatric nutritional risk index was calculated using the following formula: GNRI = 1.489 × albumin (g/L) + 41.7 × (current weight/ideal body weight). Ideal body weight was derived from the Lorentz equations (12).

The RCRI consists of six components, each assigned a binary score of 0 (absent) or 1 (present): history of ischemic heart disease; history of congestive heart failure; history of cerebrovascular disease; insulin-dependent diabetes mellitus; creatinine > 2 mg/dL; and high-risk surgery (suprainguinal vascular, intraperitoneal, or intrathoracic procedures) (13). The total RCRI score was calculated as the sum of these components.

### Outcomes

The primary outcome was a composite of PCEs, including all-cause death, resuscitated cardiac arrest, myocardial infarction, heart failure, and stroke, occurring intraoperatively or during postoperative hospitalization. Cardiac arrest was defined as the loss of circulation requiring chest compressions, defibrillation, or both (14). Myocardial infarction was defined as acute myocardial injury with clinical evidence of acute myocardial ischemia, diagnosed based on a rise or fall in cardiac troponin values (at least one value above the 99th percentile upper reference limit) accompanied by one or more of the following: symptoms of myocardial ischemia; new ischemic ECG changes; development of pathological Q waves; imaging evidence of new loss of viable myocardium or new regional wall motion abnormality consistent with ischemia; and identification of a coronary thrombus by angiography or autopsy (15). Cardiac biomarkers were assessed only when myocardial infarction was clinically suspected or ischemic ECG changes were observed. Heart failure was diagnosed based on clinical symptoms or physical examination findings, including orthopnea, dyspnea, jugular venous distention, peripheral edema, third heart sound, rales, or chest X-ray evidence of pulmonary edema or vascular redistribution (16). Stroke was diagnosed by a neurologist based on new neurological deficits confirmed by imaging (17).

### Statistical analysis

Data were systematically entered into Microsoft Excel (Microsoft, Redmond, Washington) and analyzed using Statistical Package for Social Sciences (SPSS, version 23, IBM, Armonk, New York). Date distribution was assessed using histograms and Q–Q plots. Continuous variables were summarized as median (interquartile range, IQR) or mean ± standard deviation (SD), depending on their distribution. Categorical variables were presented as frequencies and percentages. Group comparisons were performed using the Kruskal–Wallis rank-sum test or variance test for continuous variables, depending on the distribution, and the chi-squared test or Fisher’s exact test for categorical variables, as appropriate. Univariate and multivariate logistic regression analyses were conducted to identify predictors associated with outcomes. Restricted cubic spline (RCS) curves were generated using R software (version 4.2.2) with the “ggplot2” and “rcs” packages, based on logistic regression models. The optimal GNRI cutoff value was determined by the Youden index. Model performance was evaluated using receiver operating characteristic (ROC) curves, with the area under the curve (AUC) as a measure of discrimination. The DeLong test was used to compare AUC values between models. Calibration was assessed using Hosmer-Lemeshow test and calibration plots. Decision curve analysis (DCA) was performed to evaluate the clinical utility of the model. Statistical parameters, including odds ratios (OR) and 95% confidence intervals (CI), were reported. A two-tailed *p*-value < 0.05 was considered statistically significant for all analyses.

## Results

### Baseline characteristics

**Figure 1:**
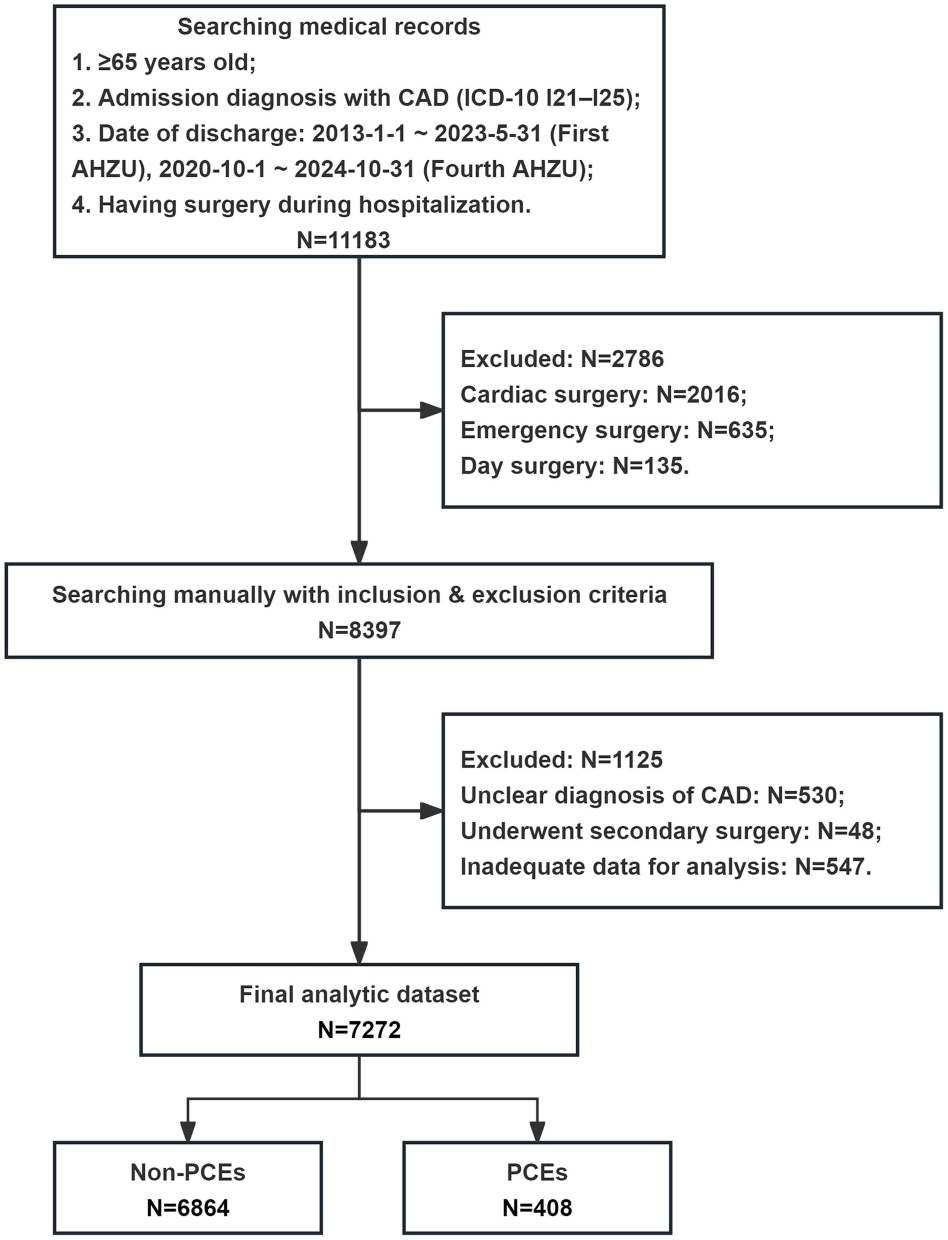
Flow chart of the patient enrollment and analysis. CAD, coronary artery disease; ICD-10, International Classification of Diseases, Tenth Revision; AHZU, Affiliated Hospital of Zhejiang University School of Medicine; PCE, perioperative cardiovascular event.

A total of 7,272 patients aged ≥ 65 years with CAD undergoing non-cardiac surgery were included in this study, with a median age of 73 years (IQR, 69–78). Figure 1 illustrates the patient enrollment and analysis flowchart. Baseline clinical characteristics and their association with perioperative outcomes are comprehensively presented in Table 1.

**Table 1:** Baseline clinical characteristics and their association with perioperative outcomes.

Variables	Total (*n* = 7,272)	Non-PCEs (*n* = 6,864)	PCEs (*n* = 408)	*P*-value
Age (years)	73 [69, 78]	73 [69, 78]	76 [70, 80]	<0.001
Male	4,764 (65.5)	4,459 (65.0)	305 (74.8)	0.001
Body mass index (kg/m^2^)	23.52 [21.37, 25.69]	23.60 [21.46, 25.71]	22.31 [19.92, 24.38]	<0.001
Diabetes mellitus	2022 (27.8)	1881 (27.4)	141 (34.6)	0.002
Hypertension	4,863 (66.9)	4,581 (66.7)	282 (69.1)	0.321
Stroke	715 (9.8)	655 (9.5)	60 (14.7)	0.001
COPD	234 (3.2)	224 (3.3)	10 (2.5)	0.366
Dialysis	82 (1.1)	59 (0.9)	23 (5.6)	<0.001
Ischemic heart disease	3,000 (41.3)	2,751 (40.1)	249 (61.0)	<0.001
Myocardial infarction	1,448 (19.9)	1,352 (19.7)	96 (23.5)	0.060
Heart failure	414 (5.7)	339 (4.9)	75 (18.4)	<0.001
Atrial fibrillation	443 (6.1)	381 (5.6)	62 (15.2)	<0.001
Valvular heart disease	112 (1.5)	95 (1.4)	17 (4.2)	<0.001
Coronary angioplasty	1799 (24.7)	1,687 (24.6)	112 (27.5)	0.191
CABG	136 (1.9)	127 (1.9)	9 (2.2)	0.606
Leukocyte (×10^9^/L)	6.1 [5.0, 7.5]	6.1 [5.0, 7.4]	6.9 [5.3, 9.6]	<0.001
Hemoglobin (g/L)	130 [116, 141]	131 [117, 142]	106 [86, 126]	<0.001
Platelet (×10^9^/L)	191 [155, 234]	192 [156, 234]	178 [134, 233]	<0.001
Creatinine (μmol/L)	78 [65, 94]	77 [65, 93]	91 [70, 132]	<0.001
Albumin (g/L)	41.8 [37.9, 44.9]	42.0 [38.3, 45.0]	37.0 [32.6, 40.7]	<0.001
ASA class				<0.001
II	2,873 (39.5)	2,803 (40.8)	70 (17.2)	
III	4,343 (59.7)	4,036 (58.8)	307 (75.2)	
IV	56 (0.8)	25 (0.4)	31 (7.6)	
Types of surgery				
General	2,110 (29.0)	1934 (28.2)	176 (43.1)	<0.001
Abdominal	1,663 (22.9)	1,505 (21.9)	158 (38.7)	<0.001
Non-abdominal	447 (6.1)	429 (6.3)	18 (4.4)	0.133
Thoracic	858 (11.8)	832 (12.1)	26 (6.4)	<0.001
Orthopedic	1,051 (14.5)	980 (14.3)	71 (17.4)	0.081
ENT	140 (1.9)	136 (2.0)	4 (1.0)	0.153
Neurological	265 (3.6)	240 (3.5)	25 (6.1)	0.006
Gynecologic	135 (1.9)	134 (2.0)	1 (0.2)	0.013
Urologic	1,276 (17.5)	1,239 (18.1)	37 (9.1)	<0.001
Ophthalmology	601 (8.3)	601 (8.8)	0 (0.0)	<0.001
Vascular	706 (9.7)	641 (9.3)	65 (15.9)	0.001
Dental	130 (1.8)	127 (1.9)	3 (0.7)	0.099
General anesthesia	5,227 (71.9)	4,898 (71.4)	329 (80.6)	<0.001
RCRI	1 [0, 2]	1 [0, 2]	2 [1, 3]	<0.001
GNRI	106 [99, 113]	107 [100, 113]	96 [87, 105]	<0.001

Results presented as median [IQR], or *n* (%).

PCE, perioperative cardiovascular event; COPD, chronic obstructive pulmonary disease; CABG, coronary artery bypass graft; ASA, American Society of Anesthesiologists; ENT, ear, nose, and throat; RCRI, revised cardiac risk index; GNRI, geriatric nutritional risk index.

Patients underwent diverse surgical procedures at two tertiary referral centers, predominantly comprising general, urologic, orthopedic, thoracic, and vascular surgeries.

The PCEs occurred in 408 patients, representing a prevalence rate of 5.6%. Compared to patients without PCEs, those with PCEs were significantly older (median age: 76 vs. 73 years, *p* < 0.001), had lower body mass index (median body mass index: 22.31 vs. 23.60 kg/m^2^, *p* < 0.001), and included more males (74.8% vs. 65.0%, *p* = 0.001). The PCEs group demonstrated significantly higher prevalence of comorbidities: diabetes mellitus (34.6% vs. 27.4%, *p* = 0.002), stroke (14.7% vs. 9.5%, *p* = 0.001), dialysis (5.6% vs. 0.9%, *p* < 0.001), ischemic heart disease (61.0% vs. 40.1%, *p* < 0.001), heart failure (18.4% vs. 4.9%, *p* < 0.001), atrial fibrillation (15.2% vs. 5.6%, *p* < 0.001), and valvular heart disease (4.2% vs. 1.4%, *p* < 0.001). ASA classification distributions also differed significantly, with the PCEs group having higher proportions of ASA III (75.2% vs. 58.8%; *p* < 0.001) and ASA IV (7.6% vs. 0.4%; *p* < 0.001) patients.

Preoperative laboratory analysis revealed significant differences between groups. The PCEs group had elevated leukocyte counts and creatinine levels but lower hemoglobin levels, platelet counts, and albumin concentrations compared to the non-PCEs group.

Regarding surgical characteristics, the PCEs group had higher rates of general anesthesia use (80.6% vs. 71.4%, *p* < 0.001) and were more likely to undergo general abdominal (38.7% vs. 21.9%, *p* < 0.001), neurological (6.1% vs. 3.5%, *p* = 0.006), and vascular surgeries (15.9% vs. 9.3%, *p* = 0.001) compared to the non-PCEs group.

### Perioperative outcomes

A total of 408 patients experienced PCEs. Heart failure was the most prevalent complication (58.6%, *n* = 239), followed by myocardial infarction (54.2%, *n* = 221), while all-cause mortality occurred in 16.4% (*n* = 67) of cases. The detailed composition of PCEs is provided in Table 2.

**Table 2:** Composition of PCEs.

PCEs	*N*	Proportion in PCEs (%)	Cumulative incidence in the entire cohort (%)
All-cause death	67	16.4	0.9
Resuscitated cardiac arrest	4	1.0	0.1
Myocardial infarction	221	54.2	3.0
Heart failure	239	58.6	3.3
Stroke	33	8.1	0.5
Total PCEs	408	100.0	5.6

PCE, perioperative cardiovascular event.

### Association between GNRI and perioperative outcomes

**Figure 2:**
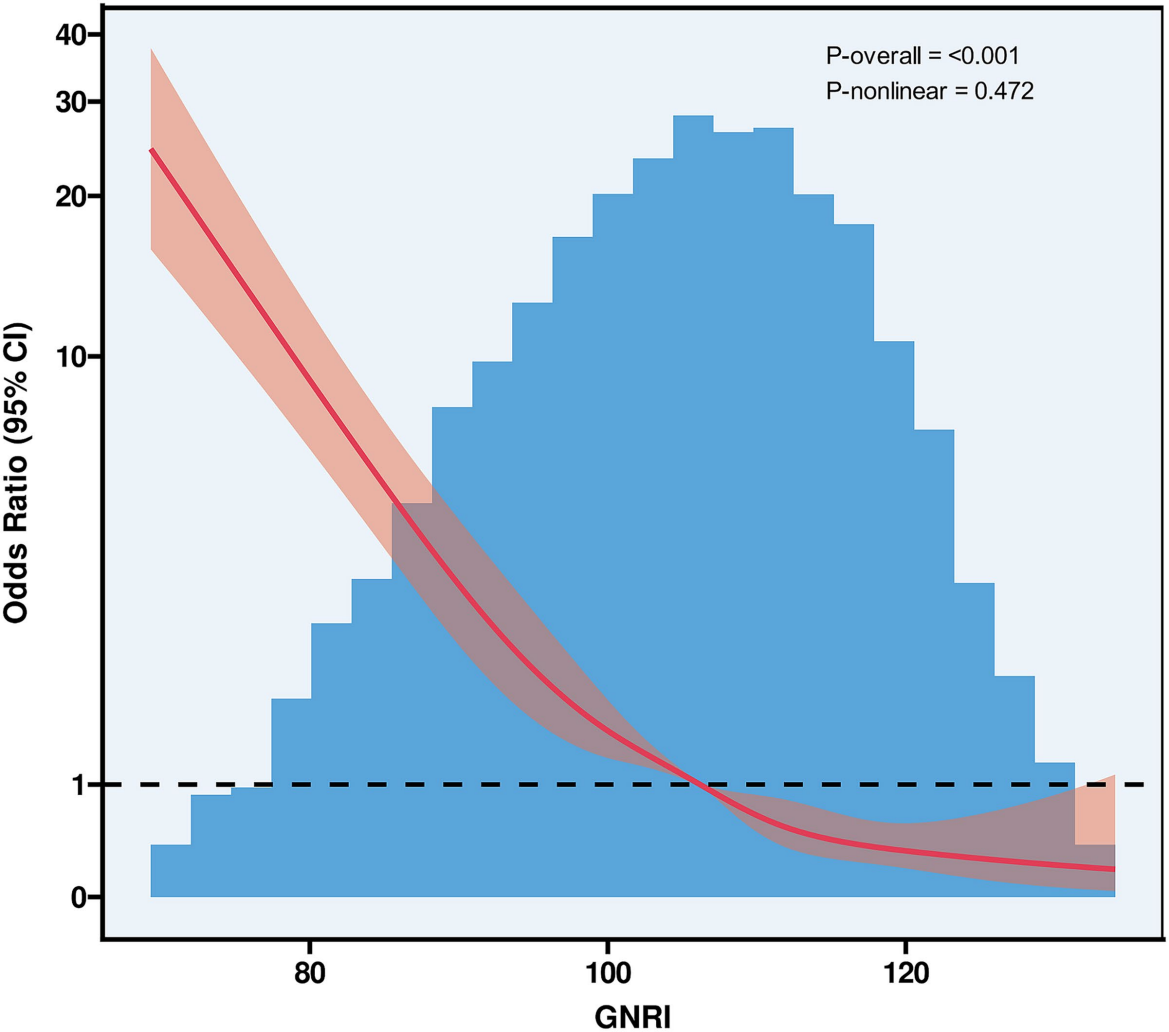
Restricted cubic spline curves of GNRI. GNRI, geriatric nutritional risk index, CI, confidence interval.

The GNRI was significantly lower in the PCEs group compared to the non-PCEs group (median GNRI: 96 vs. 107, *p* < 0.001), as detailed in Table 1. RCS curves with five knots at the 5th, 28th, 50th, 72th, and 95th percentiles were used to model the association between GNRI and PCEs (Figure 2). The RCS analysis revealed a significant inverse linear correlation between GNRI levels and PCEs (OR = 0.92; 95% CI: 0.91–0.93; *p* < 0.001).

Using the Youden index-derived optimal cutoff value (GNRI = 98), the cohort was stratified into two groups: at-risk group (GNRI < 98) and no-risk group (GNRI ≥ 98). Threshold effect analysis was performed for each group (Table 3). Univariate and multivariate regression analyses were conducted to evaluate the association between at-risk GNRI and perioperative outcomes (Table 4). Univariate analysis identified potential predictors, while multivariate analysis, after adjusting for confounders, confirmed the independent predictive value of at-risk GNRI (OR = 1.919; 95% CI: 1.496–2.461; *p* < 0.001).

**Table 3:** Threshold effect analysis of GNRI on perioperative outcomes.

Analysis method	OR (95% CI)	*P*-value
Fitting by standard logistic regression model	0.92 (0.91, 0.93)	<0.001
Fitting by piecewise logistic regression model (break-point = 98)		
GNRI < 98	0.91 (0.90, 0.93)	<0.001
GNRI ≥ 98	0.93 (0.92, 0.95)	<0.001
Log likelihood ratio		0.132

OR, odds ratio; CI, confidence interval; GNRI, geriatric nutritional risk index.

**Table 4:** Univariate and multivariate analysis of the association between at-risk GNRI and perioperative outcomes.

Analysis method	OR	95% CI	*P*-value
Model 1 (univariate analysis)	4.840	3.947–5.935	<0.001
Model 2 (preoperative patient-related covariates adjusted)	2.112	1.652–2.699	<0.001
Model 3 (surgery-related covariates adjusted)	4.044	3.281–4.984	<0.001
Model 4 (fully adjusted)	1.919	1.496–2.461	<0.001

OR, odds ratio; CI, confidence interval. Model 1 was a univariate crude model. Model 2 included age, sex, diabetes mellitus, hypertension, stroke, chronic obstructive pulmonary disease, dialysis, ischemic heart disease, myocardial infarction, heart failure, atrial fibrillation, valvular heart disease, coronary angioplasty, coronary artery bypass graft, leukocyte, hemoglobin, platelet, creatinine, and American Society of Anesthesiologists classification. Model 3 included types of surgery, and general anesthesia. Model 4 included all the confounders.

### The novel composite prognostic index

Table 5 presents univariate and multivariate analyses of GNRI and RCRI associations with perioperative outcomes. Using the multivariate regression coefficients, a weighted scoring system was developed, assigning two points to GNRI and one point to each RCRI component. This integration created a novel composite prognostic index, the GNRI plus RCRI model, with a total possible score of 8 points (2 points from GNRI and 6 points from RCRI).

**Table 5:** Univariate and multivariate analyses of GNRI and RCRI associations with perioperative outcomes.

Variables	Events	Univariate regression		Multivariate regression	
	% (*n*/*N*)	OR (95% CI)	*P*-value	OR (95% CI)	*P*-value
GNRI					
≥98	3.2 (179/5608)	Reference		Reference	
<98	13.8 (229/1664)	4.840 (3.947, 5.935)	<0.001	4.058 (3.286, 5.011)	<0.001
RCRI components					
History of ischemic heart disease					
No	3.7 (159/4272)	Reference		Reference	
Yes	8.3 (249/3000)	2.341 (1.908, 2.873)	<0.001	1.986 (1.603, 2.459)	<0.001
History of congestive heart failure					
No	4.9 (333/6858)	Reference		Reference	
Yes	18.1 (75/414)	4.335 (3.299, 5.697)	<0.001	2.640 (1.962, 3.552)	<0.001
History of cerebrovascular disease					
No	5.3 (348/6557)	Reference		Reference	
Yes	8.4 (60/715)	1.634 (1.228, 2.175)	0.001	1.329 (0.978, 1.805)	0.069
Insulin-dependent diabetes mellitus					
No	4.9 (346/6993)	Reference		Reference	
Yes	22.2 (62/279)	5.489 (4.067, 7.426)	<0.001	3.775 (2.700, 5.279)	<0.001
Creatinine > 2 mg/dL					
No	4.9 (293/6028)	Reference		Reference	
Yes	9.2 (115/1244)	1.994 (1.592, 2.497)	<0.001	1.680 (1.320, 2.138)	<0.001
High-risk surgery					
No	4.5 (201/4494)	Reference		Reference	
Yes	7.5 (207/2778)	1.720 (1.408, 2.101)	<0.001	1.687 (1.361, 2.091)	<0.001

OR, odds ratio; CI, confidence interval, GNRI, geriatric nutritional risk index; RCRI, revised cardiac risk index.

**Figure 3:**
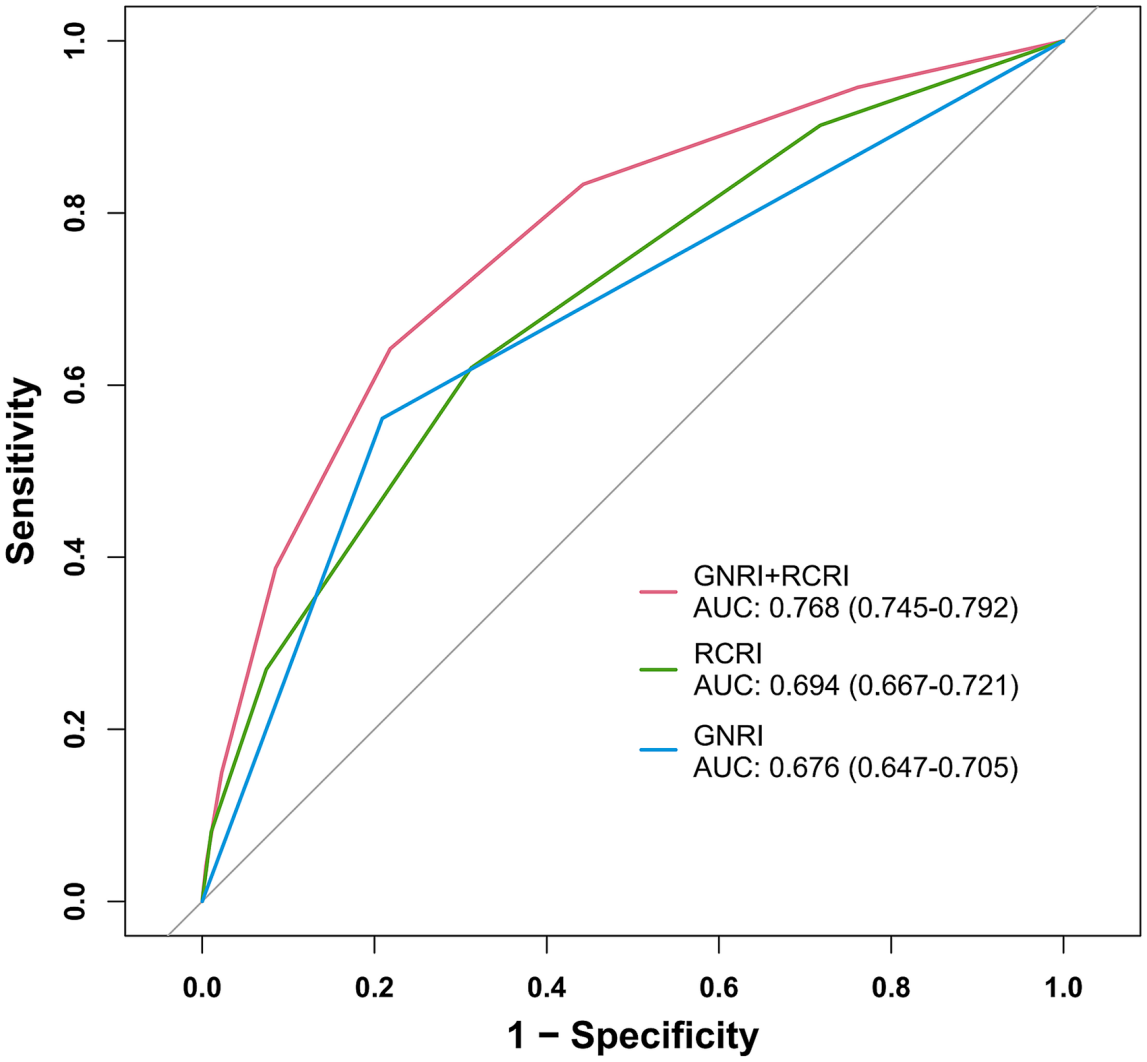
Receiver operating characteristic curves for GNRI, RCRI, and the composite model. GNRI, geriatric nutritional risk index; RCRI, revised cardiac risk index; AUC, area under the curve.

**Figure 4:**
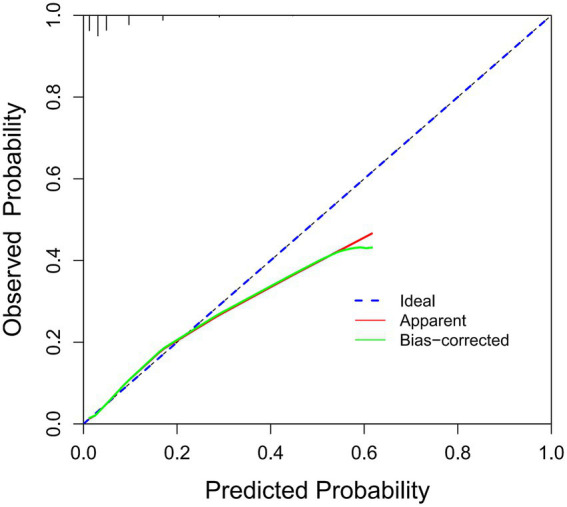
Calibration curve of the GNRI plus RCRI model. GNRI, geriatric nutritional risk index; RCRI, revised cardiac risk index.

**Figure 5:**
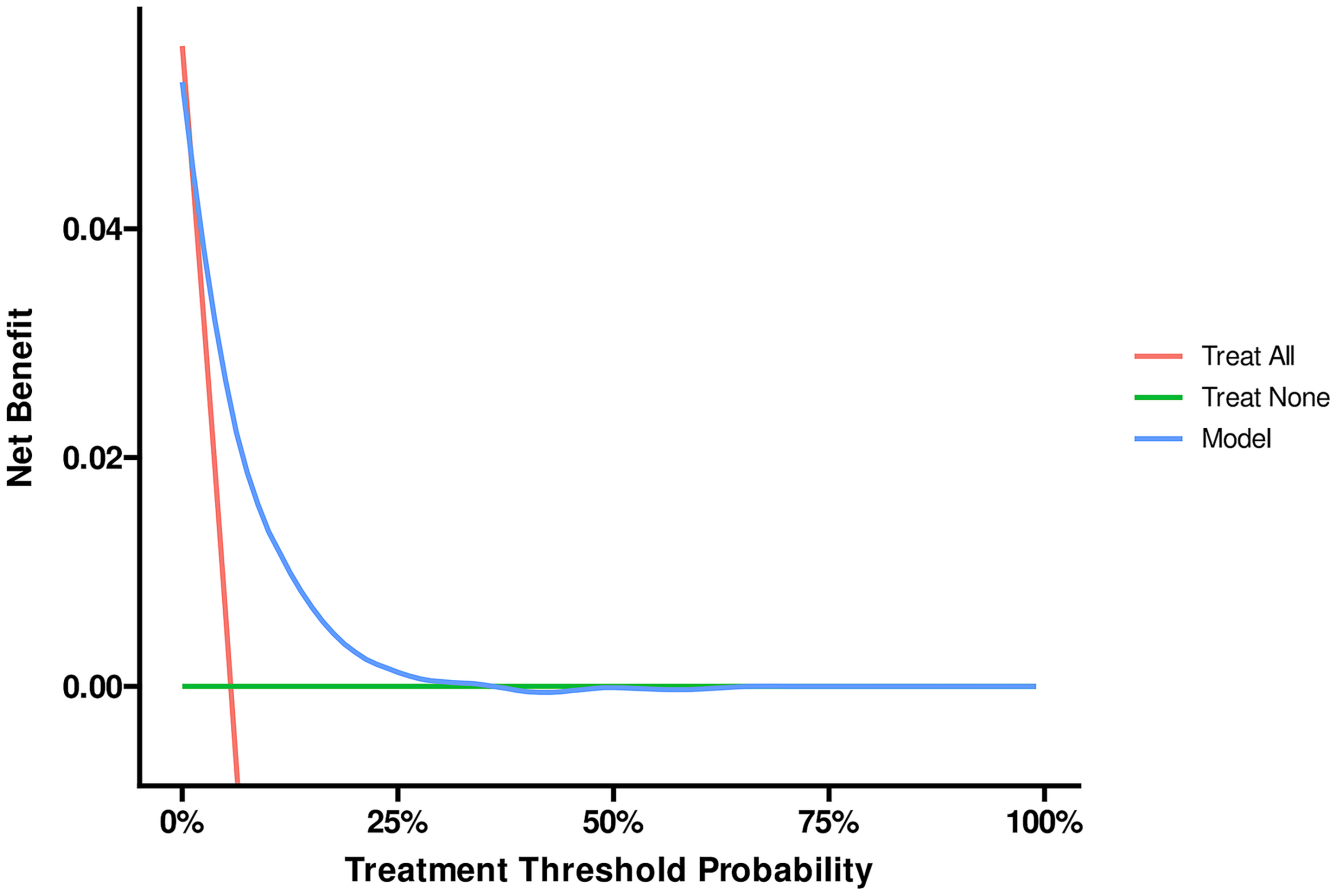
Decision curve analysis of the GNRI plus RCRI model. GNRI, geriatric nutritional risk index; RCRI, revised cardiac risk index.

The ROC analysis was used to evaluate the discriminatory ability of GNRI, RCRI, and the composite model (Figure 3). While GNRI demonstrated comparable discriminatory ability to RCRI (AUC: 0.676 vs. 0.694, *p* = 0.309), the GNRI plus RCRI model significantly outperformed both individual indices (vs. RCRI: AUC 0.768 vs. 0.694, *p* < 0.001; vs. GNRI: AUC 0.768 vs. 0.676, *p* < 0.001). The composite model exhibited good calibration, as indicated by a non-significant Hosmer-Lemeshow test (*p* = 0.391) and agreement between predicted and observed probabilities in the calibration curve (Figure 4). Decision curve analysis confirmed the superior clinical utility of the GNRI plus RCRI model across a wide range of threshold probabilities (Figure 5).

### Subgroup analysis

**Figure 6:**
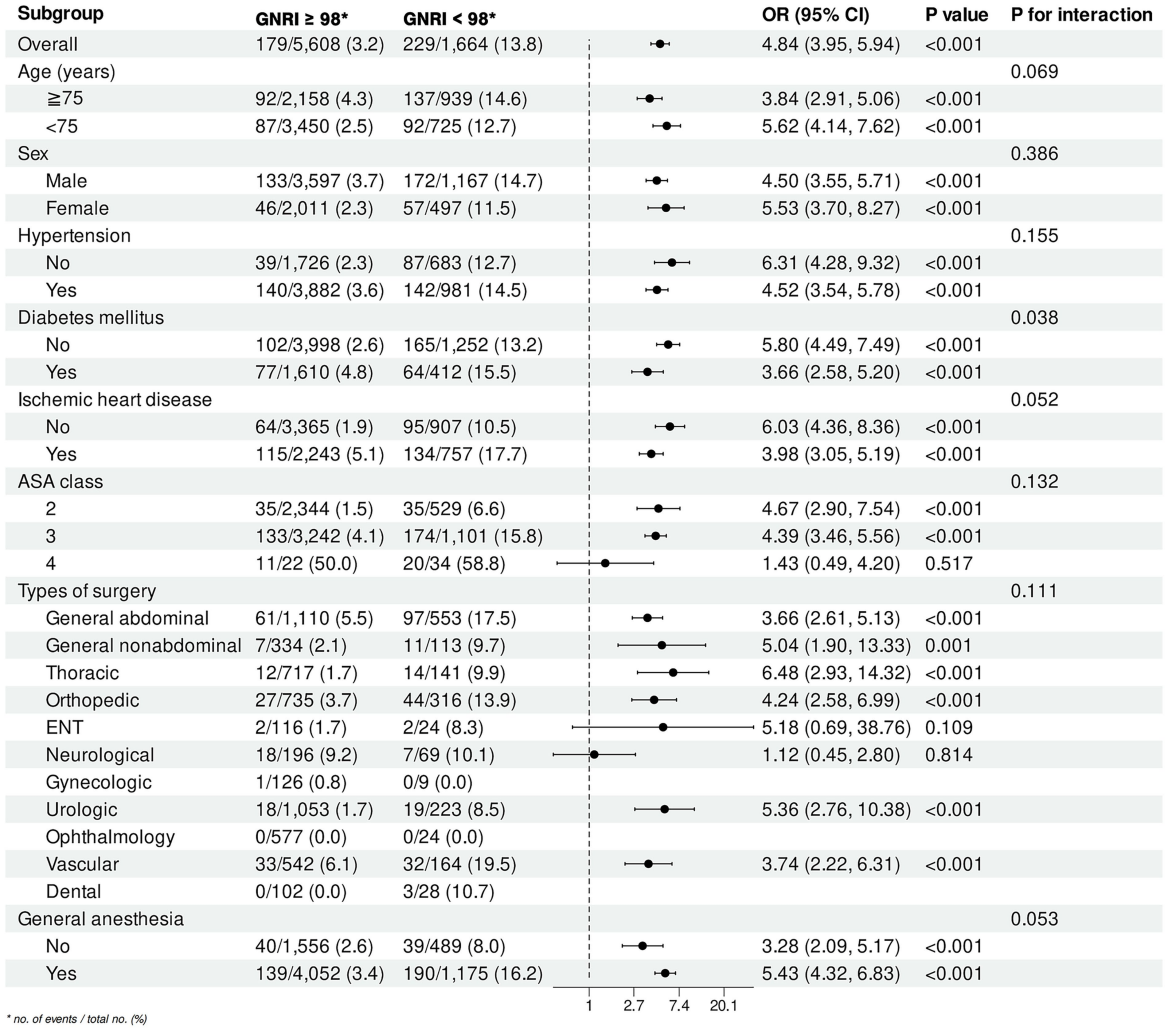
Subgroup analysis of the association between at-risk GNRI and perioperative outcomes. GNRI, geriatric nutritional risk index; OR, odds ratio; CI, confidence interval; ASA, American Society of Anesthesiologists; ENT, ear, nose, and throat.

Subgroup analysis revealed significant associations between at-risk GNRI and perioperative outcomes across various subgroups (Figure 6). Stratified by age, the interaction was not significant (*P* for interaction = 0.069). Sex-specific analysis showed no significant interaction (*P* for interaction = 0.386), with ORs of 4.50 (95% CI: 3.55–5.71, *p* < 0.001) for males and 5.53 (95% CI: 3.70–8.27, *p* < 0.001) for females. Hypertension did not significantly modify the association (*P* for interaction = 0.155), with ORs of 6.31 (95% CI: 4.28–9.32, *p* < 0.001) for non-hypertensive and 4.52 (95% CI: 3.54–5.78, *p* < 0.001) for hypertensive individuals. Diabetes mellitus showed a significant interaction (*P* for interaction = 0.038), with higher ORs in non-diabetic (OR = 5.80, 95% CI: 4.49–7.49, *p* < 0.001) compared to diabetic patients (OR = 3.66, 95% CI: 2.58–5.20, *p* < 0.001). Ischemic heart disease approached significance (*P* for interaction = 0.052), with ORs of 6.03 (95% CI: 4.36–8.36, p < 0.001) for non-ischemic and 3.98 (95% CI: 3.05–5.19, *p* < 0.001) for ischemic cases. ASA class did not significantly interact (*P* for interaction = 0.132). Among surgical types, thoracic surgery showed the highest OR (6.48, 95% CI: 2.93–14.32, p < 0.001), with no significant interaction (*P* for interaction = 0.111). General anesthesia showed a borderline significant interaction (P for interaction = 0.053), with ORs of 3.28 (95% CI: 2.09–5.17, *p* < 0.001) for non-general anesthesia and 5.43 (95% CI: 4.32–6.83, *p* < 0.001) for general anesthesia.

## Discussion

In this multicenter retrospective cohort study, we investigated the association between GNRI and PCEs in hospitalized patients aged ≥ 65 years with documented CAD undergoing non-cardiac surgery at two tertiary academic medical centers. Our findings demonstrated that GNRI was independently associated with PCEs, with consistent significance across various subgroups. The predictive performance of GNRI was statistically equivalent to the established RCRI. Moreover, integrating GNRI with RCRI enhanced perioperative risk stratification and management in this high-risk population.

This study represents the first demonstration of GNRI as a significant predictor of PCEs specifically in CAD patients. Patients with CAD constitute a high-risk surgical cohort, demonstrating a greater than two-fold increased incidence of PCEs compared to the general surgical population (18–21). Preoperative cardiovascular risk assessment is critical for this high-risk population. Although RCRI remains the most widely used risk stratification tool (22), its performance has proven suboptimal for CAD patients (5). In our study, the original RCRI demonstrated poor discriminatory ability in predicting PCEs in older CAD patients, potentially due to its inclusion of cerebrovascular disease history as a component. These findings demonstrate the urgent need for innovative risk stratification approaches incorporating novel biomarkers to optimize preoperative cardiovascular assessment in this vulnerable population.

Growing evidence underscores the prognostic significance of preoperative nutritional status across surgical specialties. However, common malnutrition screening tools, such as the malnutrition universal screening tool, may be unsuitable for routine clinical use due to complex measurement procedures and the need for professional assistance (23). In contrast, GNRI can be easily calculated using routinely measured parameters—serum albumin concentration, height, and weight—making it a practical screening tool for nutritional status in clinical settings (24). Accumulating evidence demonstrates that reduced GNRI values consistently predict adverse postoperative outcomes across surgical specialties, including 30-day mortality following bladder cancer (25) and emergency surgery (26), 180-day mortality after hip surgery (27), and 1-year mortality post-pancreatectomy (28). Consistent with prior evidence, our study demonstrates that GNRI maintains robust predictive validity for PCEs in CAD patients undergoing non-cardiac surgery. These findings underscore the clinical utility of incorporating GNRI into existing preoperative cardiovascular risk assessment protocols for this high-risk population.

In this study, GNRI exhibited a significant inverse linear correlation with PCEs. For clinical practicality, we stratified the cohort into at-risk (GNRI < 98) and no-risk (GNRI ≥ 98) groups using the optimal cutoff value determined by the Youden index. This threshold value (GNRI = 98) aligns with previously established criteria (29–31). Multivariate analysis confirmed GNRI as an independent predictor of PCEs (OR = 1.919), with discriminatory performance comparable to RCRI. Notably, while statistically significant, the standalone predictive capacity of GNRI and RCRI is relatively limited (AUC < 0.70). Therefore, we developed a weighted scoring system integrating GNRI and RCRI, which significantly improved predictive accuracy for PCEs compared to either index alone, providing a clinically practical tool for risk assessment.

Furthermore, subgroup analyses revealed significant effect modification by diabetes mellitus, with borderline significant interactions for ischemic heart disease and general anesthesia. The discriminative capacity of GNRI was weaker in patients with either diabetes mellitus or ischemic heart disease compared to those without these comorbidities. This diminished predictive performance may stem from the GNRI’s reliance on weight loss and hypoalbuminemia—both well-established independent predictors of PCEs (4, 22). Notably, these metabolic alterations occur more frequently in patients with diabetes mellitus and ischemic heart disease, likely reflecting chronic metabolic stress and inflammation (32–34). The elevated baseline prevalence of these GNRI components in these subgroups may dilute the index’s effect size, thereby reducing its discriminatory power and explaining its attenuated predictive validity. Conversely, poor nutritional status (hypoalbuminemia/weight loss) was more strongly independently associated with PCEs in patients receiving general anesthesia than those not receiving it (35). This heightened association may stem from reduced hemodynamic resilience in malnourished individuals, making them less tolerant to the physiological perturbations induced by general anesthetics (3). Accordingly, GNRI demonstrated enhanced discrimination in this subgroup.

The strengths of this study include its novelty as the first investigation of the relationship between GNRI and PCEs in CAD patients and the use of a large cohort to assess this association. Our findings indicated that preoperative at-risk GNRI was independently associated with increased PCEs compared to no-risk GNRI. Importantly, the integration of GNRI with RCRI could optimize preoperative evaluation for CAD patients. These results, supported by existing literature, advocate for the inclusion of preoperative GNRI assessment in Enhanced Recovery After Surgery (ERAS) protocols for older CAD patients undergoing non-cardiac surgery. Currently, preoperative nutritional support is not a standard component of ERAS protocols (36). Implementing GNRI as a biomarker could help identify malnourished patients at higher risk for PCEs, enabling targeted preoperative nutritional optimization.

However, this study has several limitations. First, the retrospective design of this study may introduce potential biases, including missing data and variability in perioperative nutritional therapies (enteral and parenteral). Second, although conducted across two centers, the findings may lack universal generalizability. Third, the definition of “older” is evolving; while we defined older patients as those aged ≥ 65 years, the increasing lifespan and growing population of patients aged ≥ 75 years or older necessitate similar analyses in older subgroups. Encouragingly, our subgroup analysis of patients aged ≥ 75 years yielded consistent conclusions. Fourth, our assessment of PCEs was limited to the in-hospital period, excluding post-discharge events. Consequently, the follow-up duration was relatively short, which precludes evaluation of the potential association between at-risk GNRI status and long-term survival outcomes. Future studies with extended follow-up periods are warranted to investigate this important clinical question. Fifth, the low incidence of PCEs resulted in significant class imbalance within our dataset, particularly affecting low-risk surgical subgroups. Most notably, our ophthalmology cohort exhibited complete absence of PCEs, consistent with the inherently low cardiovascular risk profile of ophthalmic procedures (37), though potential selection bias cannot be excluded. Given this limitation, the current study was unable to sufficiently assess the association between at-risk GNRI status and PCEs in ophthalmic surgery patients. To address this gap, future multicenter studies with larger, more diverse patient cohorts are needed to establish the generalizability of GNRI’s predictive value across all surgical specialties, including low-risk procedures. Finally, while we validated the predictive value of GNRI and developed a composite model to enhance preoperative evaluation, external validation is required to confirm these findings.

## Conclusion

Our study demonstrated that GNRI was independently associated with PCEs in older patients with CAD undergoing non-cardiac surgery. Incorporating GNRI into clinical decision-making may enhance perioperative risk stratification and management in this high-risk population. However, these findings warrant further validation through large-scale, multicenter prospective studies involving more diverse patient cohorts and extended follow-up periods to strengthen their generalizability and clinical applicability. Additionally, we recommend integrating preoperative GNRI assessment into ERAS protocols to optimize perioperative care for older CAD patients.

## Data Availability

The datasets presented in this article are not readily available because privacy or ethical restrictions. Requests to access the datasets should be directed to Yunpeng Jin, 8013013@zju.edu.cn.
